# Trauma exposure and the mediating role of posttraumatic stress on somatic symptoms in civilian war victims

**DOI:** 10.1186/s12888-018-1680-4

**Published:** 2018-04-10

**Authors:** Naser Morina, Ulrich Schnyder, Richard Klaghofer, Julia Müller, Chantal Martin-Soelch

**Affiliations:** 1 Department of Consultation-Liaison-Psychiatry and Psychosomatic Medicine, University Hospital, University of Zurich, Culmannstrasse 8, 8091 Zurich, Switzerland; 2 Psychiatric Services Thurgau, 8596 Münsterlingen, Switzerland; 3 Paracelsus Medical University, Salzburg, Austria; 4 Division of Clinical and Health Psychology, Department of Psychology, University of Fribourg, 1700 Fribourg, Switzerland

**Keywords:** Trauma, PTSD symptom cluster, Active avoidance, Numbing, Hyperarousal, Somatic symptoms, Civilian, War, Victims

## Abstract

**Background:**

It has been well documented that the exposure to war has a negative effect on the psychological health of civilian. However, little is known on the impact of war exposure on the physical health of the civilian population. In addition, the link between trauma exposure and somatic symptoms remain poorly understood. This cross-sectional study examined levels of somatic symptoms in the aftermath of war, and the mediating role of posttraumatic stress symptoms in the relationship between trauma exposure and somatic symptoms.

**Methods:**

Civilian war survivors (*N* = 142) from Kosovo were assessed for potentially traumatic events, posttraumatic stress symptoms, and somatic symptoms. Data were analyzed using mediation analyses. Posttraumatic stress disorder (PTSD) symptoms were categorized based on King’s four factor model (Psychol Assessment. 10: 90-96, 1998).

**Results:**

Participants reported on average more than 5 types of traumatic exposure. The cut-off indicative for PTSD was exceeded by 26.1% of participants. Symptom levels of PTSD were associated with somatic symptoms. The relationship between trauma exposure and somatic symptoms was partly mediated by the active avoidance and hyperarousal symptom clusters of PTSD.

**Conclusion:**

Active avoidance and hyperarousal symptoms seem to play a key role in traumatized people suffering from somatic symptoms.

## Background

Civilian populations exposed to trauma, such as military persecution and war, often suffer from a variety of psychological complaints including anxiety, depression, and posttraumatic stress symptoms. For instance, prevalence rates of 48% and 42% for mood and anxiety disorders, respectively, were found in Kosovar civilian adults having survived the war [1].

Somatization symptoms, i.e., somatic symptoms for which no or no sufficient organic causes are found, are frequently found to be related with posttraumatic stress disorder (PTSD), regardless of the traumatic event [2]. For example, in war veterans, combat stress reactions and PTSD were related to somatization symptoms, i.e. pain [3, 4]. Similar findings were found among a sample of factory accident survivors, where PTSD severity was positively correlated with somatization [5]. Disaster survivors suffering from PTSD reported more physical symptoms than those without PTSD [6], a finding that was also found 14 years after the genocide in Ruanda [7]. Another study found in a refugee population an association of somatization with PTSD severity [8]. Andreski et al. [9] found in their prospective study of PTSD and somatization, that PTSD increased the risk of developing somatization symptoms but that new PTSD cases were not elevated in people with a somatization disorder. Taken together, it seems that somehow PTSD may mediate the development of somatization following a traumatic event [10–12].

Various studies have examined the role of the different PTSD clusters on somatic symptoms. As such, studies have found that specific PTSD symptoms, e.g., hyperarousal may cause repeated muscle tension that could result in somatization complaints [13]. In other studies intrusive re-experiencing symptoms [6] and numbing [14] also predicted somatic symptoms in PTSD patients. There are various theoretical models to explain the association between posttraumatic stress and somatization health symptoms. For example, the shared vulnerability [15, 16] and mutual maintenance model [17], and a recent extension, the perpetual avoidance model [18], propose that individuals with PTSD and somatization symptoms have shared vulnerability to both conditions, or alternatively, that pain symptoms and posttraumatic stress symptoms interact with each other.

While there is a considerable amount of literature addressing survivors of combat, motor vehicle accidents and interpersonal trauma, war and postwar civilian populations have been less studied to date [19, 20]. To the best of our knowledge, no studies have examined war-traumatized civilians in terms of the differential relationship of each posttraumatic stress symptom cluster and somatic problems. This cross-sectional study aimed at investigating the relationship between trauma, posttraumatic stress symptoms and somatization symptoms in postwar civilians, i.e., Kosovar civilians more than a decade after the war. We hypothesized that despite the relatively long time lapse since the end of war, participants would suffer from clinically relevant symptoms of posttraumatic stress and somatization symptoms, and that specific PTSD symptom clusters would be associated with somatization symptoms.

## Methods

### Participants and procedure

The study was approved by the ethics committee of the Canton Zurich, Switzerland. Because no ethics committee was available in Kosovo, the study was approved by the Ministry of Education of Kosovo. A random sample of adult civilians from the general population of three different regions of Kosovo who had been exposed to potentially traumatic events (PTE) during the war in 1998/1999 was surveyed 10 years afterwards. Participants were included if they had been born within the territory of Kosovo; were adults, e.g. above 18 years old; had experienced at least one war-related PTE. People were excluded if they had not been residing permanently in Kosovo during the war or if they had executed higher military functions during the war. Interviews were conducted on a one-to-one verbal administration, in which the instructions and questions were read out to the participants in their native language by master-level students of clinical psychology. The details of sampling techniques and characteristics of the sample have been described in a previous publication [21].

### Measures

To pursue semantic equivalence to the original measures, the Albanian translations were conducted in accordance with the standardized translation and back-translation procedure [22]. Natively Albanian-speaking health professionals conducted the translation. The measures were tested in a pilot phase, for which 5 participants were randomly selected and then the first author, whose native language is Albanian and a native Albanian-speaking psychiatrist compared source and back-translation and adapted the translations where necessary.

### Traumatic events and PTSD symptoms

Exposure to various types of potentially traumatic events was assessed using a measure derived from combining the trauma event list of the Harvard Trauma Questionnaire [23], and the first part of the Posttraumatic Diagnostic Scale (PDS; [26]), resulting in a total of 23 items. Participants indicate if a given potentially traumatic event was experienced and/or witnessed. Subsequently, participants’ overall trauma exposure is established by summing up the number of types of trauma they had been exposed to. The severity of 17 PTSD symptoms according to the Diagnostic and Statistical Manual of Mental Disorders 4th edition (DSM-IV; [24]) criteria was assessed using the PDS [25, 26]. The frequency of each symptom in the last month was rated on a 4-point scale ranging from 0 = “*never*” up to 3 = “*5 times per week or more/nearly always*”), yielding a total severity score ranging from 0 to 51. Besides the assessment of symptom levels, the PDS allows making a categorical probable diagnosis of PTSD according to DSM-IV criteria, which requires at least one symptom of re-experiencing, three of avoidance, and two of hyperarousal. The PDS has demonstrated good psychometric properties [26]. The internal consistency in the current sample was α = .96.

To take into account the changes of PTSD in the DSM 5 the symptoms of PTSD in this sample were subdivided into four factors according to King and colleagues emotional numbing model of PTSD [27]. The model was first tested in treatment-seeking male veterans resulting in four correlated factors of Re-experiencing, Avoidance, Numbing, and Hyperarousal and was confirmed in other trauma-related populations, such as refugees [28].

### Somatization symptoms

Somatic symptoms were assessed using the 12-item somatization subscale of the Symptom Checklist-90-revised (SCL-90-R; [29]). The SCL-90-R has been translated in many languages and used in different cultural contexts [30, 31]. Each of the 12 symptoms was assessed for the last week on a 5-point Likert scale ranging from 0=“*not at all*” to 4 = “*extremely*” (range: 0–48). Items scored as 2 = “*moderately”* or above are considered as positive responses, yielding a total count of somatization symptoms. The somatization subscale in our study demonstrated good internal consistency (Cronbach’s alpha = .90).

### Statistical analysis

All analyses were conducted using SPSS 23 (SPSS inc., Chicago, IL, USA). There were less than 5% missing data on any of the variables included in the analyses. Descriptive characteristics were calculated for all outcome variables and are given in terms of means (M) and standard deviations (SD) in continuous variables, and counts and percentages in categorical variables. The mediating role of posttraumatic stress in the relationship between trauma and somatization symptoms was investigated by a statistical mediation analysis approach developed by Preacher and Hayes [32, 33]. The indirect method of this approach uses bootstrapping, which includes random resampling from the original data, with 5’000 pseudo bootstrap samples being generated. Following this, point estimates for specific and total indirect effects for each sample are provided, which then can be used to calculate indirect effects and their confidence intervals (95% CIs). The CIs are then used to define if the effects are statistically significant at *p* < .05. The use of bootstrap models is especially suggested for small sample sizes [34]. We performed a model for somatization symptoms as outcome variable, where trauma exposure was an independent variable, and the mediator variables were the four sub clusters of posttraumatic stress symptoms (re-experiencing, active avoidance, numbing and hyperarousal). All analyses were controlled for gender, age, education and employment status.

## Results

### Sample characteristics

Approximately half of the 142 assessed participants were female (*n* = 80, 56.4%). Mean age of the participants was 43.8 years (SD = 6.12), education was 10.4 years of education (SD = 3.6), and 35% were working at least part time or undergoing vocational training, whereas the remaining 65% were not employed.

### Trauma exposure and symptom severity

Participants had experienced or witnessed a mean of 5.3 (SD = 4.2) types of potentially traumatic events (see Table 1). The most frequently reported PTE’s were: “*lack of shelter” (51.4%), “lack of food or water*” (44.4%), “*being close to death*” (39.9%) and “*combat situation*” (35.9%). The least frequently reported lifetime traumatic events included sexual violence (between 1.4% and 2.1%).

**Table 1:** Trauma exposure reported by *N* = 142 civilian war survivors in post-war Kosovo region

Trauma type	*n*	%
Lack of shelter	73	51.4%
Lack of food or water	63	44.4%
Being close to death	56	39.4%
Combat situation	51	35.9%
Ill health without access to medical care	51	35.9%
Natural disaster	46	32.4%
Serious accident, fire or explosion	45	31.7%
Life-threatening illness	45	31.7%
Serious physical injury	41	28.9%
Forced separation from family member	40	28.2%
Murder of one or more strangers	36	25.4%
Unnatural death of a family member or friend	35	24.6%
Murder of a family member or friend	34	23.9%
Enforced isolation from others	23	16.2%
Torture	21	14.8%
Non-sexual assault by a stranger	21	14.8%
Imprisonment	19	13.4%
Disappearance or kidnapping	14	9.9%
Non-sexual assault by a family member or someone you know	11	7.7%
Brainwashing	6	4.2%
Sexual assault by a family member or someone you know	3	2.1%
Sexual assault by a stranger	2	1.4%
Sexual contact when you were younger than 18 with someone who was 5 or more years older than you	2	1.4%

Notes: Potentially traumatic events

The PDS cut-off score for probable PTSD was exceeded by 26.1% of participants. Participants with probable PTSD and no PTSD did not differ on socio-demographic variables (age: *p* = .195; gender: *p* = .406).

The severity score for somatization symptoms on the SCL-90-R was 10.3 (SD = 8.5) Participants reported on average 3.3 (SD = 3.3) different somatization symptoms with a range of 0 to 12.

Women had higher levels of somatization symptoms (*p* = .004) than men, whereas levels of PTSD symptoms were similar between men and women (*p* = .290). Participants with a probable diagnosis of PTSD showed higher scores on somatization symptoms (see Table 2).

**Table 2:** Somatization severity and symptom count by PTSD diagnosis (*N* = 142)

	PTSD (*n* = 37)		No PTSD (*n* = 105)		t-test
	M	SD	M	SD	
Somatization Severity	15.9	8.8	8.3	7.5	5.104***
Somatization Symptom Count	5.65	3.38	2.57	2.29	−4.130***

*Notes*: Somatization: subscale Somatization SCL-90-R, PTSD PTSD in posttraumatic diagnostic scale

***p < .001

### Relationship between PTSD and somatization symptoms

**Fig. 1:**
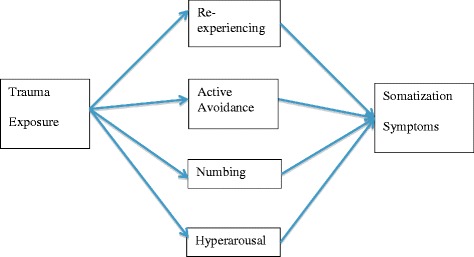
Model of mediation analyses investigating the relationship between trauma, posttraumatic stress symptoms and somatization outcomes

The results from the mediation analysis are presented in Table 3 (see Fig. 1 for diagrammatic representation of the tested model). According to the mediation analyses, trauma exposure was significantly associated with somatization symptoms. Trauma exposure was significantly associated with posttraumatic re-experiencing, active avoidance, numbing and hyperarousal. With respect to the effect of posttraumatic stress symptoms (mediator) on somatization symptoms (outcome), only active avoidance and hyper-arousal were significantly associated with somatization symptoms. Posttraumatic stress thus partly mediated the relationship between trauma exposure and somatization symptoms, accounting for 37% of the variance in somatization symptoms. There was a significant indirect negative effect of posttraumatic stress symptoms on somatization symptoms via active avoidance and a significant indirect positive effect of posttraumatic stress symptoms via hyperarousal.

**Table 3:** Results of analyses examining the mediating role of PTSD symptoms on the relationship between trauma exposure and somatization symptoms

		DV - Somatization Symptoms				
Independent variable (*IV*)	Mediating variable (*MV*)	Direct effects (c’)	Total effects (c)			
Trauma		.45**	.62***	Adj. R^2^ = .37, F(9,132) = 10.36, *p* < .001		
		Effect of *IV* on *MV* (*a*)	Effect of *MV* on *DV* (*b*)	Indirect effects (*a x b*)	95% CI Low	95% CI High
Trauma	Re-experiencing	.28**	.40	.115	−.044	.367
Trauma	Active Avoidance	.16***	−.91*	−.150*	−.360	−.036
Trauma	Numbing	.18**	.19	.035	−.097	.284
Trauma	Hyperarousal	.24***	.68**	.166*	.011	.358

Notes: * *p* < 0.05; ** *p* < 0.01; *** *p* < 0.001. Re-experiencing = Re-experiencing subscale of PTSD in PDS; Active Avoidance = Avoidance subscale of PTSD in PDS; Numbing = Numbing subscale of PTSD in PDS; Hyperarousal = Hyperarousal subscale of PTSD in PDS; Somatization: Subscale Somatization SCL-90-R

## Discussion

In this cross-sectional study, we examined the relationship between trauma exposure, posttraumatic stress symptoms and the level of somatization symptoms in a randomly selected sample of Kosovar civilian war survivors (*N* = 142). Substantial trauma exposure and high levels of clinically relevant symptoms of PTSD and somatization symptoms were found. Somatization symptoms correlated significantly with PTSD symptoms. In mediation analyses, the relationship between traumatic exposure and somatization symptoms was partly mediated by active avoidance and by posttraumatic hyperarousal, accounting for 37% of the variance in somatization symptoms.

As expected, compared to non-conflict countries, lifetime trauma exposure was substantial in the civilian population in Kosovo [35–37]. This is unsurprising since the cohort was sampled from a war-affected region. Further, the high rates of probable PTSD (26%) and the high levels of somatization symptoms in the present sample are in line with earlier studies conducted in the post-war Balkans [1, 38, 39].

In addition, consistent with findings in the previous literature, female gender was associated with higher scores on somatization symptoms [40–42]. However, PTSD in the present study was equally found in women and men, which is in contrast to the epidemiological literature, but has been shown in samples of military personnel [43]. There is evidence, though, that gender may play a mediating role in the relationship of trauma and somatization symptoms, with females developing more somatization complaints as compared to men [9, 40]. This pattern overlaps with the robust finding of more females developing PTSD than males [44].

One of the interesting findings in this study was the differential role of PTSD symptom clusters on somatization symptoms. Specifically, we found that arousal mediated the association between trauma exposure and somatization symptoms using the PTSD cluster’s classification by King and colleagues [27]. A comparable result was found by McFarlane et al. [6] in a sample of fire-fighters. The present study adds to the previous literature, however, by subdividing the avoidance cluster into active avoidance and numbing to the model. It is also conceivable that the numbing symptoms overlap with alexithymia and depressive symptoms, which have been shown to mediate the association of trauma exposure on somatization in major depression [45]. In addition, it is possible that passive avoidance may contribute to somatization presentations, as shown in the Escalona and Colleagues study [14], because these responses can include social and emotional withdrawal, which in turn might increase awareness of and focus on internal sensory perception. Avoidance behaviors in people suffering from pain can be attributed, in part, to catastrophizing about the severity of the pain as a predominant somatic symptom and the patient’s inability to function, which may contribute to fear of pain and lead to avoidance of activities [46].

Active avoidance is a mediator of the relationship between trauma and somatic symptoms in a negative direction. This means the more one avoids situations and thoughts that remind him of the trauma, the less somatization symptoms one has. Further, this could mean, if someone is avoidant of thoughts and feelings, he is not very good at recognizing how he feels and so reports lower levels of subjective somatic symptoms. There has also been considerable attention given to the bidirectional relationship between PTSD and posttraumatic somatization symptoms such as pain. As Sharp and Harvey (Sharp & Harvey, 2001) have argued, PTSD patients can have their trauma memories triggered by episodes of pain because their pain is associated with the experience of the traumatic event. Conversely, posttraumatic stress can exacerbate pain by (a) increasing arousal, (b) elevating muscle tension, (c) promoting vigilance to pain sensations, and (d) exaggerating negative appraisals about the severity of the pain experience.

Additional war-related consequences, such as social problems, may complicate the understanding of etiology, assessment, and course of treatment. In a low-income country such as Kosovo, socio-economic hardship and health problems might additionally be associated. Beyond this, it has been suggested that among some non-western, collectivistic cultures, somatization symptoms are among the most frequent presentations of trauma survivors [47, 48]. In Kosovo, it is conceivable that being psychologically ill might be socially unacceptable, whereas presenting somatization symptoms is more acceptable in Kosovarian people.

This study has several limitations. First, our results are based on self-reports assessed by questionnaires which usually have lower reliability than structured clinical interviews. However, because questionnaires were administered within a personal interview conducted by trained clinical psychology master students, we expect sufficient reliability. Another limitation is the use of instruments that have not yet been validated in Albanian. However, translations and back-translations were thoroughly performed by experienced and natively speaking interpreters in mental health and after consensus discussions adapted by mental health care providers, if necessary, in order to achieve maximum quality. The long period between the war and our assessment might have induced inaccurate recall; moreover, it is not clear whether the problems reported by participants were a consequence of the war, the current social situation and living conditions, or other adversities participants had been exposed to during the course of their lifetime. Finally, the cross-sectional approach of the present study does not allow to draw any conclusions regarding causal relationships between trauma exposure and the presence of somatization symptoms. However, our findings allow us to generate specific hypotheses regarding possible mechanisms and causal factors contributing to the prominent somatization symptoms in traumatized civilian war survivors. These precise assumptions about etiological mechanisms linking trauma exposure to somatization symptoms must be investigated in further research with longitudinal designs.

## Conclusions

More than a decade after the end of the Kosovo war, the presence of posttraumatic stress and somatic symptoms in the civilian population exposed to war is still substantial. Symptoms of posttraumatic mental health and somatic symptoms are highly associated, indicating a complex underlying mechanism. This study suggests that specific symptoms of PTSD, namely active avoidance and hyperarousal patterns, partially mediate this association. Active avoidance was associated with less somatic symptoms, whereas hyperaraousal was relate do more somatic symptoms. Therefore, when civilian survivors of war present with somatic complaints in primary care, mental health problems should always be taken into account as a differential diagnosis. The main approach should be the development of combined or stepped care treatments in terms of a bio-psycho-social model [49]. Finally, in the future, longitudinal studies with larger sample sizes allowing for multivariate analyses should be conducted in order to investigate underlying mechanisms, predictors, and causal factors.

## Abbreviations

CI: Confidence interval
DSM-IV: Diagnostic and statistical manual of mental disorders 4th edition
M: Means
PDS: Posttraumatic diagnostic scale
PTE: potentially traumatic events
PTSD: Posttraumatic stress disorder
SCL-90-R: Symptom Checklist-90-revised
SD: Standard deviations

## References

[1] Priebe S (2010). Mental disorders following war in the Balkans: a study in 5 countries. Arch Gen Psychiatry.

[2] Sack M (2007). Trauma prevalence and somatoform symptoms: are there specific somatoform symptoms related to traumatic experiences?. J Nerv Ment Dis.

[3] Shalev A, Bleich A, Ursano RJ (1990). Posttraumatic stress disorder: somatic comorbidity and effort tolerance. Psychosomatics.

[4] Solomon Z, Mikulincer M (1987). Combat stress reactions, post traumatic stress disorder and somatic complaints among Israeli soldiers. J Psychosom Res.

[5] Elklit A, Christiansen DM (2009). Predictive factors for somatization in a trauma sample. Clin Pract Epidemiol Ment Health.

[6] McFarlane AC (1994). Physical symptoms in post-traumatic stress disorder. J Psychosom Res.

[7] Rieder H, Elbert T. The relationship between organized violence, family violence and mental health: findings from a community-based survey in Muhanga, southern Rwanda. Eur J Psychotraumatol. 2013;4. 10.3402/ejpt.v4i0.21329. 10.3402/ejpt.v4i0.21329 PMC3828565 24244834

[8] Spiller TR (2016). Somatisation and anger are associated with symptom severity of posttraumatic stress disorder in severely traumatised refugees and asylum seekers. Swiss Med Wkly.

[9] Andreski P, Chilcoat H, Breslau N (1998). Post-traumatic stress disorder and somatization symptoms: a prospective study. Psychiatry Res.

[10] Gupta MA (2013). Review of somatic symptoms in post-traumatic stress disorder. Int Rev Psychiatry.

[11] Pacella ML, Hruska B, Delahanty DL (2013). The physical health consequences of PTSD and PTSD symptoms: a meta-analytic review. J Anxiety Disord.

[12] Afari N (2014). Psychological trauma and functional somatic syndromes: a systematic review and meta-analysis. Psychosom Med.

[13] Liedl A (2010). Support for the mutual maintenance of pain and post-traumatic stress disorder symptoms. Psychol Med.

[14] Escalona R (2004). PTSD and somatization in women treated at a VA primary care clinic. Psychosomatics.

[15] Asmundson GJG, Katz J (2009). Understanding the co-occurrence of anxiety disorders and chronic pain: state-of-the-art. Depress Anxiety.

[16] Asmundson GJG (2002). PTSD and the experience of pain: research and clinical implications of shared vulnerability and mutual maintenance models. Can J Psychiatr.

[17] Sharp TJ, Harvey AG (2001). Chronic pain and posttraumatic stress disorder: mutual maintenance?. Clin Psychol Rev.

[18] Liedl A, Knaevelsrud C (2008). Chronic pain and PTSD: the perpetual avoidance model and its treatment implications. Torture.

[19] Teodorescu DS (2015). Chronic pain in multi-traumatized outpatients with a refugee background resettled in Norway: a cross-sectional study. BMC Psychol.

[20] Carlsson JM (2006). Mental health and health-related quality of life: a 10-year follow-up of tortured refugees. J Nerv Ment Dis.

[21] Schick M, et al. Trauma, mental health and intergenerational associations in Kosovar families 11 years after the war. Eur J Psychotraumatol. 2013;4. 10.3402/ejpt.v4i0.21060. 10.3402/ejpt.v4i0.21060 PMC3744842 23956820

[22] Bontempo R (1993). Translation fidelity of psychological scales: an item response theory analysis of an individualism-collectivism scale. J Cross-Cult Psychol.

[23] Mollica RF (1992). The Harvard trauma questionnaire*.* Validating a cross-cultural instrument for measuring torture, trauma, and posttraumatic stress disorder in Indochinese refugees. J Nerv Ment Dis.

[24] APA (1994). Diagnostic and statistical manual of mental disorders*: DSM-IV*.

[25] Foa EB (1995). PDS (posttraumatic stress diagnostic scale) manual.

[26] Foa EB (1997). The validation of a self-report measure of posttraumatic stress disorder: the posttraumatic diagnostic scale. Psychol Assess.

[27] King DW (1998). Confirmatory factor analysis of the clinician-administered PTSD scale: evidence for the dimensionality of posttraumatic stress disorder. Psychol Assessment.

[28] Palmieri PA, Marshall GN, Schell TL (2007). Confirmatory factor analysis of posttraumatic stress symptoms in Cambodian refugees. J Trauma Stress.

[29] Derogatis LR (1977). SCL-90-R: administration, scoring and procedure manual - I.

[30] Ventevogel P (2007). Properties of the Hopkins symptom Checklist-25 (HSCL-25) and the self-reporting questionnaire (SRQ-20) as screening instruments used in primary care in Afghanistan. Soc Psychiatry Psychiatr Epidemiol.

[31] Mollica RF (1987). Indochinese versions of the Hopkins symptom Checklist-25: a screening instrument for the psychiatric care of refugees. Am J Psychiatry.

[32] Preacher KJ, Hayes AF (2008). Asymptotic and resampling strategies for assessing and comparing indirect effects in multiple mediator models. Behav Res Methods.

[33] Hayes AF (2009). Beyond baron and Kenny: statistical mediation analysis in the new millenium. Comm Mon.

[34] Shrout PE, Bolger N (2002). Mediation in experimental and nonexperimental studies: new procedures and recommendations. Psychol Methods.

[35] Perkonigg A (2000). Traumatic events and post-traumatic stress disorder in the community: prevalence, risk factors and comorbidity. Acta Psychiatr Scand.

[36] National Institute of Mental Health. Statistics. 2012. Retrieved June 16, 2012 from: http://www.nimh.nih.gov/statistics .

[37] Kessler RC (1995). Posttraumatic stress disorder in the National Comorbidity Survey. Arch Gen Psychiatry.

[38] Kashdan TB, Morina N, Priebe S (2009). Post-traumatic stress disorder, social anxiety disorder, and depression in survivors of the Kosovo war: experiential avoidance as a contributor to distress and quality of life. J Anxiety Disord.

[39] Morina N, Ford JD (2008). Complex sequelae of psychological trauma among Kosovar civilian war victims. Int J Soc Psychiatry.

[40] McCall-Hosenfeld JS (2014). The association of interpersonal trauma with somatic symptom severity in a primary care population with chronic pain: exploring the role of gender and the mental health sequelae of trauma. J Psychosom Res.

[41] Haug TT, Mykletun A, Dahl AA (2004). The association between anxiety, depression, and somatic symptoms in a large population: the HUNT-II study. Psychosom Med.

[42] Morina N (2010). Somatic distress among Kosovar civilian war survivors: relationship to trauma exposure and the mediating role of experiential avoidance. Soc Psychiatry Psychiatr Epidemiol.

[43] Norris FH, Slone LB, Friedman MJ, Keane TM, Resick PA (2007). The epidemiology of trauma and PTSD. in Handbook of PTSD: science and practice.

[44] Olff M (2007). Gender differences in posttraumatic stress disorder. Psychol Bull.

[45] Gulec MY (2013). Effects of childhood trauma on somatization in major depressive disorder: the role of alexithymia. J Affect Disord.

[46] Vlaeyen JW, Linton SJ (2000). Fear-avoidance and its consequences in chronic musculoskeletal pain: a state of the art. Pain.

[47] Kirmayer LJ, Young A (1998). Culture and somatization: clinical, epidemiological, and ethnographic perspectives. Psychosom Med.

[48] Ford CV (1997). Somatic symptoms, somatization, and traumatic stress: an overview. Nordic Journal of Psychiatry.

[49] Otis JD (2009). The development of an integrated treatment for veterans with comorbid chronic pain and posttraumatic stress disorder. Pain Med.

